# Special Issue “Transcriptional Regulation in Plant Development: 2nd Edition”

**DOI:** 10.3390/ijms27020751

**Published:** 2026-01-12

**Authors:** Konstantinos E Vlachonasios

**Affiliations:** 1 Department of Botany, School of Biology, Faculty of Sciences, Aristotle University of Thessaloniki, 54124 Thessaloniki, Greece; kvlachon@bio.auth.gr; 2 Natural Products Research Centre of Excellence (NatPro-AUTh), Center of Interdisciplinary Research and Innovation, Aristotle University of Thessaloniki (CIRI-AUTh), 54124 Thessaloniki, Greece

## 1. Epigenetic and Transcriptional Mastery of Development

The following Special Issue highlights the sophisticated transcriptional regulatory networks governing plant development, with a specific focus on how genetic, epigenetic, and metabolic pathways integrate environmental signals to shape agronomic traits. With this mindset, we present the second edition of Transcriptional Regulation in Plant Development, which features a collection of 14 articles. From model species such as Arabidopsis to economically vital crops including coffee, lilies, and sesame, the gathered research underscores the complexity of maintaining developmental plasticity in a changing world.

We extend our sincere gratitude to the authors, peer reviewers, and the editorial team for their contributions, without which this Special Issue would not have been possible. Together, their work reflects both the depth of scientific inquiry and the collaborative effort required to address one of the most complex challenges of plant development.

The contributions can be grouped into four major themes: molecular/epigenetic regulation, phase transition and stress resilience, organelle function and quality, and hormonal regulation of stress and growth.

Central to plant morphogenesis is the precise control of gene expression. The research findings presented in this issue illuminate how GCN5-mediated histone acetylation is indispensable for floral fertility, specifically through its genetic interaction with the GA-signalling repressor RGA, which facilitates stamen elongation [1]. Similarly, the role of TCP5 transcription factors is revealed as a "Goldilocks" mechanism in anther development; both overexpression and suppression lead to male sterility, demonstrating that precise TCP5 levels are required for effective pollen sac formation and lignin-driven anther dehiscence [2].

## 2. Regulating Phase Transitions and Stress Resilience

Environmental cues are primary drivers of developmental shifts. In Lilium, the molecular “switch” for the vegetative-to-reproductive transition is identified as the Lbr-miR171b–LbrSCL6 module, which responds to temperature fluctuations to accelerate flowering [3].

Resilience to abiotic stress remains a critical focus for crop improvement. Genome-wide analyses in lettuce identified LsHSP70-19 as a vital chaperone for heat tolerance, with its overexpression significantly enhancing root and fresh weight under thermal stress [4]. In sesame, a core set of 759 waterlogging-responsive genes and key transcription factors (AP2/ERF and WRKY) was mapped, providing a blueprint for breeding flood-tolerant varieties [5].

## 3. Genomic Insights into Organelle Function and Quality

Beyond the nucleus, organellar genomes play pivotal roles in adaptation and quality. The assembly of the *Phragmites australis* mitogenome offers new insights into energy allocation and adaptive evolution in perennial grasses [6]. Conversely, mitochondrial dysfunction is identified as the primary culprit behind "spawn ageing" in *Agaricus bisporus* mushrooms, where downregulation of energy metabolism and ROS-scavenging genes limits commercial yield [7].

Lastly, the nutritional and commercial value of crops is often tied to specialised metabolic pathways. In safflower, the integration of transcriptomics and metabolomics pinpointed flavonoid synthesis genes (CHS, F3H) as the drivers of flower pigmentation [8]. Similarly, in *Coffee arabica*, the identification of the *CaFAD8* gene provides a genetic target for modulating fatty acid unsaturation, a key precursor to the volatile compounds that define coffee aroma [9].

## 4. Hormonal Regulation of Stress and Growth

Hormones act as the primary transducers of environmental stress. Research on *Populus euphratica* resulted in the identification of the PeAREB04 transcription factor as a major player in drought resistance; its overexpression enhances root elongation and reduces water loss by regulating stomatal aperture [10]. In Jerusalem artichoke, the results of a multi-omics analysis revealed that jasmonic acid and salicylic acid promote tuber sprouting by modulating fructan metabolism, illustrating a tight link between hormone signalling and carbohydrate metabolism [11].

The interplay between hormones is further elucidated in *Rhododendron chrysanthum*, where exogenous ABA mitigates UV-B damage. This protection is achieved by enhancing glutathione metabolism, modulating gibberellin (GA) levels, and altering the acetylation patterns of key antioxidant enzymes [12].

A primary theme of this issue is the identification of specific genetic loci that dictate plant architecture. In *Brassica rapa*, the discovery of the *Brcl1YS* locus (homologous to the GA-pathway regulator RGL1) provides new insights into how cell development differences lead to leaf curling [13]. In orchids (*Cymbidium*), the expansion and diversification of the *SWEET* gene family through genome duplication events have been linked to complex floral patterning and diurnal metabolic rhythms, offering a foundation for molecular breeding in ornamental horticulture [14].

## 5. Conclusions

The studies presented herein bridge the gap between fundamental molecular biology and practical agronomic management. By identifying the specific genetic regulators—from miRNAs and transcription factors to epigenetic modifiers—that respond to internal hormones and external stressors, this Special Issue provides an essential molecular resource for the next generation of crop improvement and horticultural innovation.

We hope that the discoveries and insights presented herein will inspire further innovation, foster collaboration across disciplines, and ultimately contribute to the knowledge of complex developmental traits. The second edition of this Special Issue serves not only as a collection of knowledge but also as an invitation to think boldly, challenge assumptions, and continue striving toward a future defined by crop resilience and improvement.
